# Consumption—Drinks, Food, Bathing, Exercise, Clothing and Treatment

**Published:** 1879-09

**Authors:** James H. Sallisbury

**Affiliations:** Cleveland, Ohio


### CONSUMPTION—DRINKS, FOOD, BATHING, EXERCISE, CLOTHING AND TREATMENT.

---

By JAMES H. SALLISBURY, A.M., M.D., Etc., Cleveland, Ohio.

---

I. Drinks—Drink half a pint of hot water one hour before each meal and on retiring, for the purpose of washing out the slimy, yeasty, and bilious stomach before eating and sleeping. Drink a cup of clear tea, coffee, or beef tea (the latter free from fat) at each meal, and hot water or beef tea, if thirsty.

II. Food—*Meats*—Eat broiled beef-steak, which has been entirely freed from fat and bone. Have it seasoned to taste with salt, pepper and butter. For variety, use broiled chicken, broiled game, oysters, roasted in shell or broiled, broiled lamb or mutton (lean), broiled cod-fish (fresh and salt), broiled and baked fish free from fat, and broiled dried beef, chipped thin and sprinkled over beefsteak. A soft boiled egg may be taken occasionally at breakfast with the meat, if it does not heighten the color of the urine.

*Bread.* Bread, toast, boiled rice, cracked wheat or oat meal mush may be eaten in the proportion of one part (by bulk) to from four to six parts of the meat. The bread should be free from sugar and raised with yeast. It may be made from gluten flour, white flour, or Graham flour, corn meal should be avoided.

All things not previously enumerated, and the following articles of food should not be eaten, viz.: Beans, soups, sweets, pies, cakes, pickles, sauce, preserves, fruits, vegetables, greens, pancakes, fritters, crullers, griddle cakes and mush. Vinegar should also be avoided. Use butter, pepper and salt for seasoning; also use either Worcestershire or Halford sauce, mustard and horse radish, with lemon juice on meats if desired. Celery may be moderately used as a relish.

III. Baths.—Take a soap and hot water bath twice a week for cleanliness, after which oil the entire body, rubbing in well. Every night sponge the body and limbs with one quart of hot water, in which put from one to two teaspoonfuls of aqua ammonia; after which rub well and wipe dry. Every morning, sponge off with a little hot water, wiping dry and rubbing thoroughly.

IV. Clothing.—Wear flannel next the skin, and dress comfortably warm. Change all clothing worn during the day, on retiring, so that it may be thoroughly aired for the following morning. Keep the clothing sweet and clean, by changing every other day.

V. Exercise.—Ride daily in the open air as much as possible, without fatigue. If not able to ride, the body and limbs should be rubbed and pounded all over for ten minutes morning, noon and night, by some one who has sufficient strength to do it thoroughly.

VI. Meals.—Meals should be taken at regular intervals, and it is better not to sit down to a table where others are indulging in all kinds of food. Eat alone, or with those only who are on the same kind of diet. After the system gets in good running order, which is indicated by the urine flowing at the rate of three pints in twenty-four hours, and standing constantly at 1.020 density, the appetite becomes good, and usually more than three meals a day are desired. This desire for food should be gratified by allowing the patient a nice piece of broiled steak, with a cup of clear tea, coffee, hot water, or beef tea midway between breakfast and dinner, and dinner and supper.

VII. Treatment. Before each meal, the patient should take a small dose of some good tonic. If there is a softened state of the tissues of the lungs, endangering hæmorrhage, something like the following would be a good tonic:

℞—Fluid extract pyrus malus radice.......... ℥iij
" " witch hazel.................. ℥iiss
" " cinchona comp.............. ℥iiss
" " ginger...................... ℥ss
" " yerba santa.................. ʒiij
" " grindelia robusta, comp....... ℥iiss
" " sundew...................... ℥j
" " water fennel seed............ ℥j
" " orange peel.................. ℥ss
al. menth. pip. gtt. xx.; al. winter green, gtt. xij

M. S.: Take a teaspoonful in water before each meal. If the disease is complicated with chronic diarrhœa or consumption of the bowels, something like the following would be appropriate:

℞—Fluid extract cranesbill.................. ℥iiss

" " ginger...................... ℥ss

" " witch hazel................. ℥iiss

" " pyrus malus radice.......... ℥iij

" " ampelopsis.................. ℥j

" " yerba santa................. ʒiiss

" " orange peel................. ℥ss

" " winter green................ ℥ij

" " chinchona comp............. ℥ij al. menth. pip., gtt. xv.

M. S.: Take a teaspoonful in water before each meal.

Immediately after each meal, should be given either 8 grs. of pepsin, 8 grs. of lactopeptine, or 8 grs. of pansaline. This latter remedy is composed as follows:

℞—Boudalt's pepsin..........℥j

Pancreatine..............ʒiij

Phloridzine..............ʒiij

Lactic acid..... .........ʒij

M.

To sweeten the stomach and bowels and also aid in checking diarrhœa if there is any, give:

℞—Carbolic acid (pure white crystals).....ʒss

Aqua, ℥viij, al. menth. pip..............gtt. x

M. S.: Take a teaspoonful in a little water fifteen minutes after each meal.

To assist in making blood or to tone up the enervated nervous system, the following is a very good pill:

℞—Pil. phosphorous.........(1–100 gr.)
Strychnine..............(1–100 gr.)
Iron..................... 1 gr.

S.: Take a pill two hours after breakfast or dinner.

To invigorate and to improve the condition of the mucous membranes throughout the digestive tract, and to so tone up the throat and fauces as to prevent taking cold at every slight exposure, take the following:

℞—Spts. ammonia, aromatic..............℥viij

Salicin..............................℥ss

M. S.; (Shake well before taking.) Dose—Take from one-half to one teaspoonful in a wine-glass of water one hour after each meal.

If there is constipation of the bowels, use the mildest means to stimulate their muscles and glands, so that they will move once every day about breakfast time if possible.

The following external applications will be found useful.

℞—Emplastrum belladonnæ
(Spread with the alcoholic extract.)

S. Apply to chest.

If there is danger of hæmmorrhage, provide the patient with an atomizer and a weak solution of persulphate of iron (one drachm to eight ounces of water) and instruct him to have the apparatus in readiness, so that as soon as there are indications of bleeding, to inhale at once the spray of this mixture. It will check the bleeding in a few minutes.

R—Salzburg porus plasters........two.

S. Apply one over bowels and one between the shoulders.

If the directions here given are faithfully followed out and persisted in, consumption *in all its stages* becomes a curable disease.

All anodynes, that get the stomach out of order, are to be rigidly avoided. The cure is accomplished by getting the system in splendid condition, when the urine becomes clear and flows at the rate of three pints daily—standing at 1.020 density; the appetite becomes enormous, so that from two to four pounds of nice lean beef are eaten daily with a relish.

The chills, fevers and sweats, grow lighter and lighter, and finally cease entirely ; the blood-making process goes on rapidly ; the blood vessels fill out ; repair of tissues begins and goes steadily on ; the eyes brighten ; the cough gradually grows less and less ; interstitial death, decay and disintegration of lung tissue ceases ; the glow of health pervades the entire organism, and step by step the patient (if he perseveres) advances safely and surely toward health, which to reach only requires patience and the rigid observance of the rules here laid down. To accomplish this, the diet and treatment are to be closely and conscientiously carried out in all their details, with the soul and body of the patient enlisted in the good cause. Of course it takes time ; for nature, after all does the work, and consequently all the changes must be physiological, and only as rapid as the human machine—when well run—can organize and repair. The physician must know precisely what to do, and do it. He must watch his patient daily, examining excretions, secretions and blood carefully and see that every part of the programe is faithfully and honestly carried out.

Any deviation from the right course can be detected at once, by an increase in fermentation, consequent biliousness, heightened color of urine and aggravation of cough, and all the other pathological symptoms. Patients cannot deceive the skilled physician in this field of positive work. If the directions are all rigidly followed the machine will soon get to running nicely and continue to do so, till thrown off the track by departures. These departures should be detected and corrected at once, or the patient begins to lose ground. No one need hope to handle consumption successfully simply by change of climate and medicine. It is a disease arising from continued unhealthy alimentation, and must be cured by removing the cause. This cause is fermented food and the products of this fermentation. Carbonic acid gas, alcoholic and vinegar yeast and vinegar, are the important factors in developing the peculiar pathological symptoms, conditions and states in this dangerous and generally believed incurable complaint.

Consumption of the bowels can be produced at any time, in the human subject, in from 15 to 30 days, and consumption of the lungs inside of three months by special exclusive and continued feeding upon the diet that produces them.

The foregoing are a few pages from the work on *Consumption* which I have had for sometime ready for publication. I have been treating the disease successfully for twenty years, and have had under my care during that time over one thousand cases. I have simply to say that the disease is so thoroughly worked up in all its details that I am able to produce it at will and as surely cure it. This any one can be satisfied of, by coming and watching the patients under treatment.—*Virginia Medical Monthly.*

